# Interferon in systemic lupus erythematosus—A halfway between monogenic autoinflammatory and autoimmune disease

**DOI:** 10.1016/j.heliyon.2022.e11741

**Published:** 2022-11-24

**Authors:** Ioana Saulescu, Ruxandra Ionescu, Daniela Opris-Belinski

**Affiliations:** a University of Medicine and Pharmacy Carol Davila, Dionisie Lupu Street, Number 37, Postal Code 020021, Bucharest, Romania; b Sfanta Maria Hospital, Internal Medicine and Rheumatology Department, Ion Mihalache Boulevard, Number 37-39, Postal Code 011172, Bucharest, Romania

**Keywords:** Interferon, Systemic lupus erythematosus, Toll like receptor, Interferonopathies, Autoimmunity, Autoinflammation

## Abstract

Although perceived as an adaptative immune disorder, mainly related to Lymphocyte B and T, last years focus on Systemic Lupus Erythematosus (SLE) pathogeny emphasised the important role of innate immunity. This should not take us by surprise since the lupus cell described by Hargraves and colleagues in 1948 was a neutrophil or macrophage with specific aspect after coloration with haematoxylin related to cell detritus engulfment (Hargraves et al., 1948) [1] (*Presentation of two bone marrow elements; the tart.***Hargraves M, Ricmond H, Morton R.** 1948, Proc Staff Meet Mayo Clinic, pp. 23:25–28). Normal immune system maintains homeostasis through innate and adaptative response that are working together to prevent both infection and autoimmunity. Failure of the immune mechanisms to preserve the balance between these two will initiate and propagate autoinflammation and/or autoimmunity. It is well known now that autoinflammation and autoimmunity are the two extremes of different pathologic conditions marked with multiple overlaps in many diseases. Recent findings in SLE demonstrated that innate immune system initiates the abnormal autoimmunity and starts the continuous inflammatory reaction after that, interferon being one of the key cytokines in innate immunity and SLE. Understanding this mechanism might offer a better clue for an efficient treatment in SLE patients. The purpose of this review is to highlight the enormous impact of innate immunity and mostly interferons in SLE.

## 1 Introduction

Systemic lupus erythematosus (SLE) is a complex disease characterized by autoantibodies appearance that target nuclear components, immune complexes formation and deposition and tissue inflammation that trigger organ damage. First hints about haw the disease appears are from 1948 when Hargraves and his colleagues described the lupus cell, part of the apoptotic mechanism [1]. It is well established now that genetic background, environmental—especially infectious—triggers, hormonal factors and immunological abnormality interact for the disease appearance. Patients might have a very wide spectrum of organ involvement, with variable clinical features from mild, non—threatening organ involvement like joints or skin to severe, organ threatening involvement like renal, hematologic, or central nervous system (CNS) manifestations. Differences between patients regarding the disease course also exist, varying from longstanding quiescent disease to a waxing and waning pattern in most of the cases, with irreversible damage accumulation in time [2, 3].

Nowadays, a well-defined concept is related to SLE. Disease appears in specific steps: first there is a long period of time when genetic susceptibility, hormonal background and environmental factors establish the predisposition to autoimmune process. When the immune tolerance is broken, autoantibodies against self will appear, initially the non-specific ones, followed by the most specific and then the organ involvement will start. In time, irreversible damage and comorbidities related to genetic predisposition, chronic inflammation, or therapies will become prominent [4].

Characterised as a multifactorial autoimmune disorder and with genetic polymorphism, special types of SLE like familial SLE or early-onset juvenile SLE—as monogenic forms of this pathology—made possible a better understanding of the disease pathogenesis, mainly related to innate immune response [5, 6]. This facts are in line with what last decade scientific discovery showed about immunological disorders: most rheumatic and musculoskeletal diseases (RMDs) exist as a continuum, innate immune-mediated autoinflammatory diseases representing one end of the spectrum and adaptative immune-mediated autoimmune diseases being placed at the other end [7, 8]. It is well known now that the two immune responses are working together, and that the related diseases are determined by the incorrect balance between them [9, 10].

Dysregulated functions of these two immune systems have the origin in different components and cells involved. Autoimmunity is associated mainly with malfunction of the adaptative immune response, involving T and B lymphocytes and having as a result autoantibodies production, while autoinflammation is synonym with innate immune response abnormality, being characterised by inflammatory flares and the involved cells being neutrophils, dendritic cells (DCs), or macrophages [11, 12]. Despite differences in the principal players, they resemble in important point: both have self-tissue inflammation or/and autoimmunity without a direct specific initiation factor.

It is obvious now that there is no clear line to delineate autoimmunity from autoinflammation in some of the RMDs pathologies and mostly in SLE, since some diseases embrace features from both parts. This is demonstrated both by the pathogenic mechanisms involved, but also by the clinical manifestations appearing during the disease evolution. Regarding SLE, there is plenty of similar features in the clinical picture from the autoinflammatory disorders: evolution with episodes of flare, fever, rash, serositis, interstitial lung involvement, Central Nervous System abnormalities or arthritis and this is more stringent in monogenic forms of the disease [13]. In the opposite corner, many autoinflammatory diseases may express evanescent autoimmunity with antinuclear antibodies, making differential diagnosis—especially for paediatric population—even more difficult [14, 15]. Continuum perspective upon autoimmune and autoinflammatory disorders, proper knowledge of different pathogenic mechanisms involved could offer help in daily practice. Being able to recognise implication of the innate immune response, connecting its important effectors—Interferons (IFNs) with patient clinical picture in multifactorial SLE will help the clinicians to make better choices from the treatment perspective.

## 2 Methods

A systematic search of the literature was done with the scope to capture the IFN implication in autoimmunity and autoinflammation. Medical subject headings (MeSH) terms “Type I Interferon”, “Autoinflammatory disease”, “Systemic Lupus Erythematosus” and “Innate Immunity” were used. The search was performed in PubMed, Cochrane Library and Thompson Reutersʹ Web of Science Core Collection, identifying relevant sources for this review. Titles and abstracts were screened first. When an abstract was selected, the full-text article was evaluated and, if eligible, selected to be used. Reference lists of this articles were also checked for additional records.

Articles were considered eligible if:

- Covered SLE pathogeny, including innate immune response, respectively IFN in SLE,
- Covered autoinflammatory diseases, including IFN driven pathologies,
- Referred to pathogenic mechanisms, clinical pictures, or specific treatment.

Articles were excluded in case of editorial papers, animal studies, articles not matching at least two keywords (eg SLE and innate immunity, autoinflammation and IFN), or when language used was other than English.

**Figure 1:**
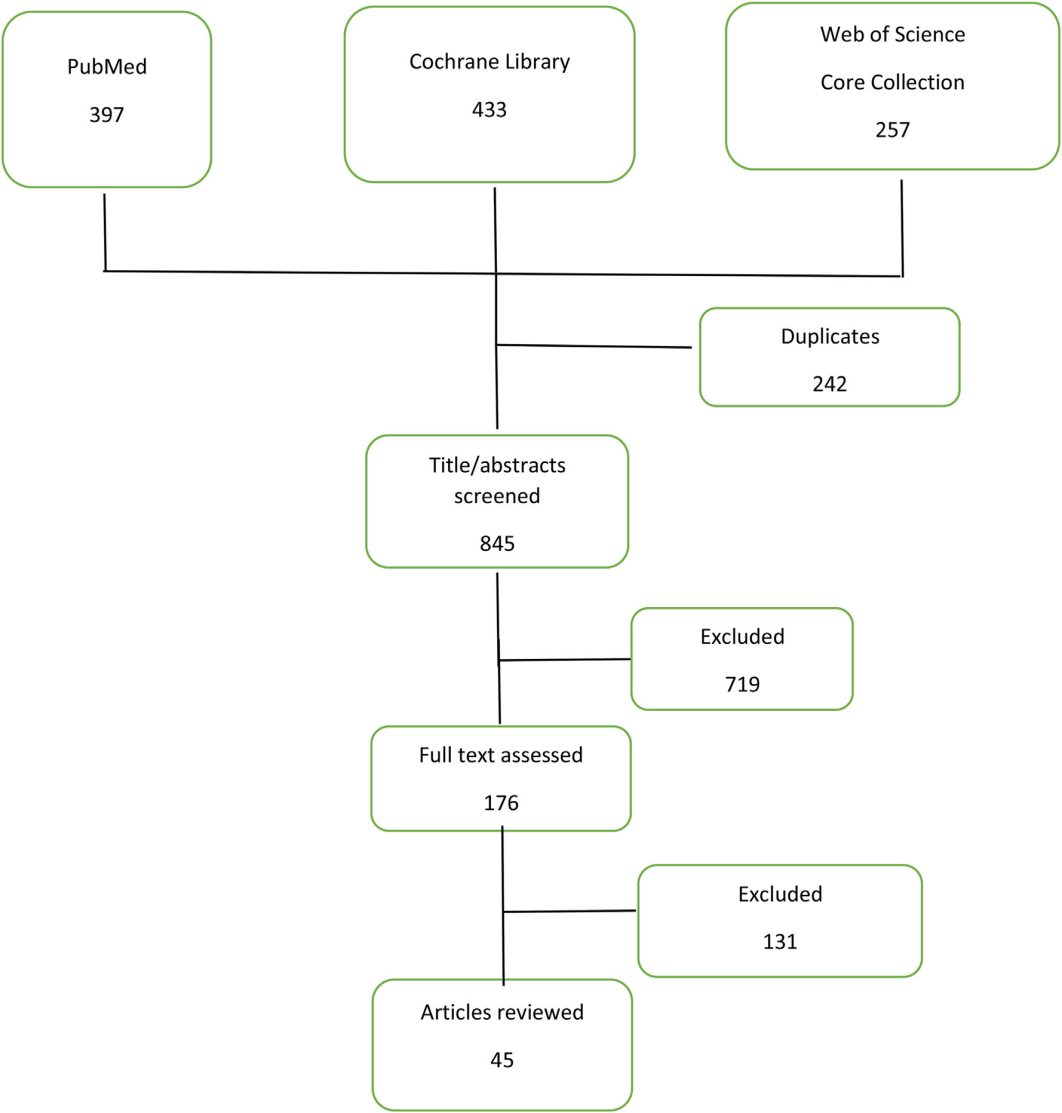
Results of systematic search and selection process.

Search strategy is pictured in Figure 1.

## 3 Pathogenic rationale: IFN perspective

### 3.1 Innate immunity—link between loss of tolerance and exaggerate inflammation

The innate immune response represents the first line of defence with a sharp reaction against microbial invasion or self-danger signals, having as a result elimination of pathogen or infected cells, activation of the specific adaptative immune response, maintenance of self-tolerance and tissue repair [16, 17]. Being present since birth, received directly from the ancestors, it is capable of action in a few minutes, starting an inflammatory reaction that is vital for protection against the unwanted when this is requested. Its role is crucial not only related to host defence, but also in conducting most of the immune interactions, being a common player in autoimmunity and autoinflammation.

Its actions are possible because of four components that are working together: the physical barriers (mainly junctions between skin cells, epithelial and mucous membrane surfaces, mucus and blood vessel endothelial cells) [16, 18, 19], the cellular compartment (neutrophils, macrophages, natural killer cells, innate lymphoid cells, mast cells, DCs), the inflammation related serum proteins (complement system, C-reactive protein and lectins) and the cytokines—important regulators for cell-cell communications, but also for controlling the inflammation, immunity and haematopoiesis—IFNs, interleukins (Il) or tumor necrosis factor (TNF)) [16, 20]. Along with these four master components other factors will work to preserve normal immune actions: antimicrobial enzymes (lysozyme), antimicrobial peptides (defensins, cathelicidins), cell receptors (toll-like receptors (TLRs)), and the inflammasome [21].

Functions of the innate immunity include first-line defence against infections, maintenance of immunologic homeostasis, and activation of adaptative immune responses. Taken all together these reflect its capability to initiate inflammatory response when needed—clinically corelated with *tumor, rubor, dolor, calor*, followed by the proper mechanisms to stop this reaction when the intruder is gone. Regarding activation of the adaptative immune response, innate mechanisms offer the possibility to a more focused reaction, preventing an immune response against a harmless environmental antigen or even to a self—antigen. In other words, innate immune response has downregulatory mechanisms to preserve or restore immune homeostasis [22].

Correct recognition of an antigen is the first step towards a well-functioning immunity [20]. Cells with phagocytic function can identify conserved structures that are on the pathogen’ surface, like viral RNA or bacterial lipopolysaccharide (pathogen—associated molecular patterns (PAMPs)), but also endogenous molecules produced or released by damaged cells (damage—associated molecular patterns (DAMPs)). These molecules are recognized by relatively non-specific patterns recognition receptors (PRRs) that are localized on the phagocytic cells. After recognition, transcription of the proinflammatory genes will start, followed by the release of the cytokines and chemokines. Induction of pyroptosis—a proinflammatory cell death will eliminate the unwanted trigger [16, 17].

Two main groups of PRRs are known: secreted or circulating proteins and peptides (defensins, cathelicidin, C1q from the complement system, collectins, lectins, pentraxins) and a more traditional ones, like transmembrane and intracellular signal-transducing receptors (TLRs, nucleotide-binding oligomerization domain (NOD)-like receptors (NLRs), RIG-1 like receptors (RLRs)) [16, 17, 23]. Membrane-bound PRRs are found on a numerous innate immune cell and on the antigen-presenting cells, including B-lymphocytes. Signalling through PRRs have the effect of a sentinel. After activation, a cascade of other PRRs is upregulated and the immune response will start. Originally viewed as a highly performant surveillance system, engaged in discrimination between pathogen and host, late years discovery in autoimmune diseases showed that PRRs have a tremendous role in starting and perpetuation this type of disorders. The same PRRs can recognize endogenous ligands that are expressed or released from dying or dead cells. In physiologic condition, response to DAMPs is important for the clearance of cell debris, but when the mechanism of control fail to stop self-recognition process, a continuous activation of the innate immune response will start [24].

Appearance of autoantibodies, cytokines, or other soluble mediators in SLE are verry similar with the normal immune response against a microbial pathogen. In both situation—SLE or infectious disease—immune response starts through innate immune cells that are found at the mechanical barriers: skin, mucous membrane. Environmental factors associated with SLE should pass one of the mechanical barriers that are part of the innate immune response: UV light—the skin; smoking, dust, allergens—respiratory mucous tract. This is where the disease begins, emphasising once again the tremendous role of the innate immunity in appearance of the disease.

A step forward was done when implication of TLRs –part of PRRs—was revealed. These receptors recognize classes of stimuli with characteristic structural features, although they are part of the non-specific innate immune response [17]. Among the TLR ligands there are also nucleic acids. These are typical components of bacteria or viruses, but the origin might be in the apoptotic process of the human host to. Regardless of the origin, activation of the TLRs will heighten the immune response, innate and adaptative, followed by cytokines and autoantibodies appearance and perpetuation [16, 17]. There are at least 10 human different TLRs implicated in both types of the immune response, and malfunction of the TLRs pathway will be associated with autoimmunity and autoinflammation [17]. An important factor regarding TLRs activity is cellular localization. Expression of TLR3, TLR7, TLR8 and TLR9 are within endosomes, while the others are localized on cell surface. TLR7 and TLR9 are both implicated in the immune process of SLE. Abnormal TLR7 signalling is related to RNA structure, while TLR9 is associated with DNA material. Increased levels of microparticles that contain DNA and RNA, nuclear proteins, growth factor or cytokines are found in SLE patients, being released during inefficient apoptosis and it can start inflammatory process and autoimmunity [16, 17]. Activation of TLRs will lead to phosphorylation of different transcription factors, including the one for IFN, a potent link between inflammation and autoantibody production.

### 3.2 Interferon: major link between autoimmunity and autoinflammation

Described for the first time 20 years ago, autoinflammatory disease practically changed the understanding of the pathogenies of different conditions, starting with atherosclerosis and cardiovascular disease and culminating with autoimmune diseases like SLE. This is now known as “autoinflammatory revolution” and had an important impact for understanding different immune-mediated pathologies [25].

Suggestion that IFN could have a role in SLE pathogenesis came from the observational studies of cancer patients treated with IFN-α that developed lupus-like disease, including antinuclear antibodies (ANA) [26, 27]. Today, abnormal IFN pathway is associated with multifactorial autoimmune disease like SLE and monogenic autoinflammatory diseases like interferonopathies or monogenic SLE as captured in Table 1, demonstrating common pathogenic mechanisms and suggesting possible common treatment approaches.

**Table 1:** Continuum spectrum of Interferon immune mediated diseases.

Disease	Type	Genetic defect	Cytokine	Treatment	References
Systemic lupus erythematosus (SLE)	Rheumatic autoimmune/autoinflammatory disease	Multifactorial	IFN	Corticosteroids HCQ Immunosuppressants Biologics (anti B cell, anti IFN)	2, 4, 5
Aicardi Goutieres syndrome	Autoinflammatory disease	Monogenic	IFN	JAK inhibitors	16, 26, 71
Sting- Associated Vasculopathy with onset in Infancy (SAVI)	Autoinflammatory disease	Monogenic	IFN	JAK inhibitors	16, 26, 71
Monogenic SLE	Autoinflammatory disease	Monogenic	Subtypes with IFN	JAK inhibitors	16, 26, 71

At the beginning, IFN was only viewed as a protein capable to “interfere” with viral replication [28]. Part of the innate defence mechanism, it has a major role in fighting against viral infections, large amounts of IFN being released when the virus encounters the host, inhibition of viral replication being the result [26].

There are three different types of IFN. Type I family is the largest and most studied in relation with aberrant function of the immune response. This family is formed by five classes, IFNα being divided in 13 subtypes [26, 29]. Type I IFNs is produced by most cells, but the principal producer is represented by the plasmacytoid dendritic cell (pDC). There is only one type II IFN, produced mostly by T and Natural Killer (NK) cells. Type III IFNs is a family of four with abundant expression at barrier surfaces, being released by epithelial and epithelial-origin cells, including macrophages and DCs [26, 29, 30, 31].

At this moment, type I IFN family is known for its implication in immune disorders. These type of IFN has an immunomodulator effect, shaping the innate immune response and activating the adaptative one, in order to protect human body. It also has an antitumoral surveillance role with antiproliferative consequence [29]. Its production is normally induced by viruses, bacteria, or microbial nucleic acids via recognition through PRRs. All type I IFNs bind to the same ubiquitously transmembrane receptor—IFNAR, composed of IFNAR1 and IFNAR2. When type I IFN binds to these receptors, activation of Janus kinase (JAK) 1 and tyrosine kinase (TIC) 2 pathway will start, followed by the formation of the interferon-stimulated gene factor 3-complex (IGSF3), including signal transducer and activator of transcription (STAT) 1, STAT 2, and interferon regulatory factor (IRF) 9. IGSF3 will further promote IFN—regulated genes [29, 32, 33] responsible for action on both innate and adaptative immune response.

Type I IFNs are produced in small amounts by a multitude of cells, like monocytes, neutrophils, or keratinocytes, but pDCs are known as natural interferon-producing cells since they are capable to produce a large amount of IFN—α in response to many different micro—organisms. This explains the importance of pDCs in SLE pathogenesis. Although they exist in small number in peripheral blood mononuclear cells, they are recruited to the inflammation sites where they start to produce IFN, being found in skin or kidney of the patients with SLE. It is postulated now that pDCs are responsible for the persistent secretion of IFN in lupus patients [26, 27]. Targeting pDCs could represent a way to decrease the expression of IFN response genes, ameliorating inflammatory lesions of these patients.

Triggers for IFN production might be UV irradiation, infections (mainly viral), injury or cell death. These triggers are responsible for appearance of DAMPs and PAMPs, signalling IFN production via PRR [26, 29]. In SLE, UV light and infectious contribute both to the appearance of the disease in a genetic predisposed host, but they can also initiate a disease flare [26].

The fact that patients with SLE have an increased level of IFN was described long before genome-wide expression analysis become available, showing that 50%–70% of adult patients and up to 90% of paediatric SLE population have an increased expression of type I IFN—regulated genes, known as IFN signature. IFN activity is more intense in younger patients and in more active disease [34, 35].

Being a multifactorial disease, studies of genome-wide expression analysis in SLE showed existence of more than 100 genetic risk loci and more than half of them encodes proteins that are directly or indirectly linked to type I IFN production or functions [26, 36, 37]. These include genes related to TLRs activation or signalling molecules or risk loci associated with STAT 4 known to determine a particular phenotype of SLE with appearance of the disease at an early age with severe renal and neurological involvement. It is well demonstrated now that STAT 4 risk allele needs to interact with other gene or environmental factors to become pathogenic, being postulated that prolonged exposure to type I IFN is necessary in order to develop SLE [26, 38].

**Figure 2:**
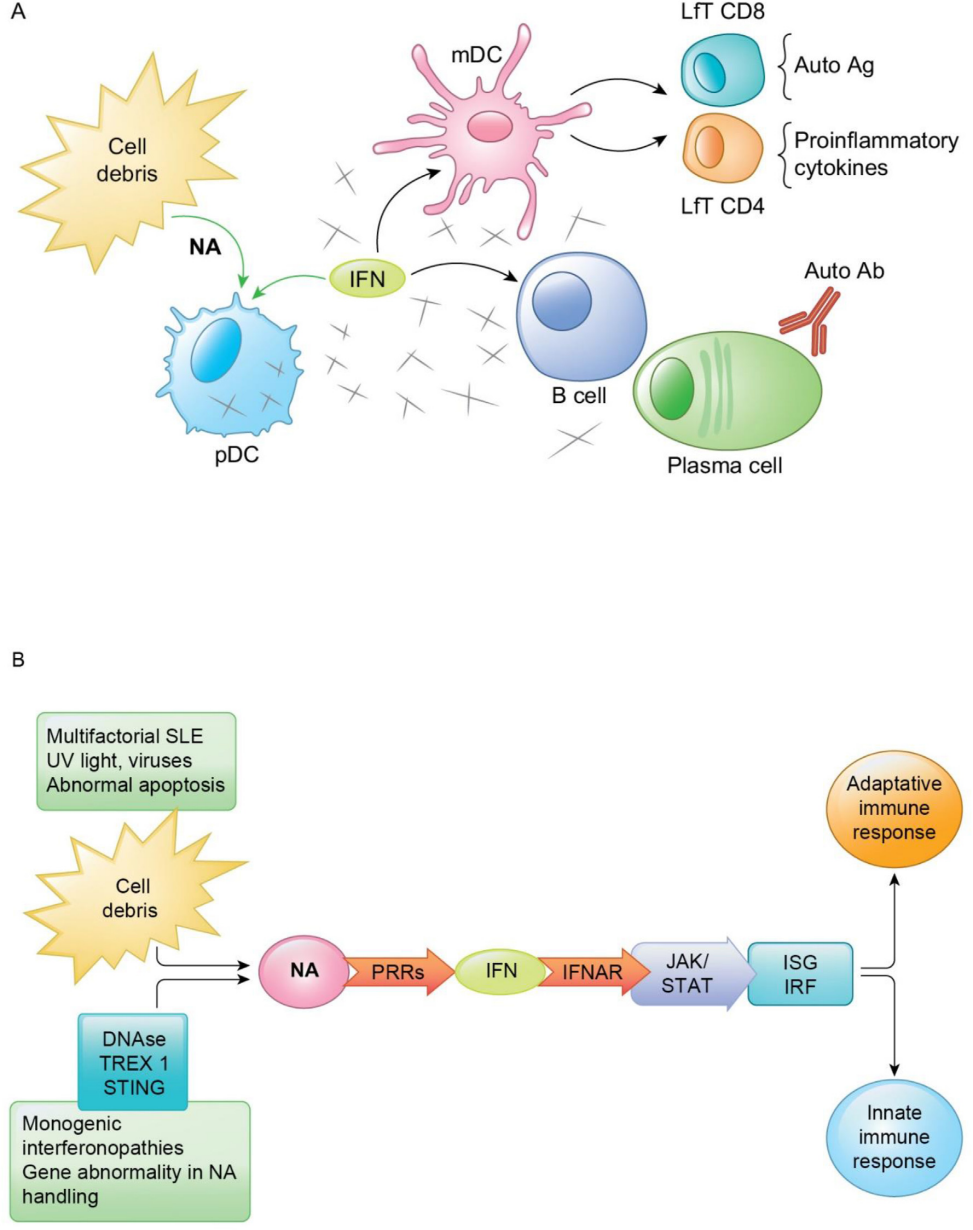
A. Schematic representation of Interferon (IFN) perspective in SLE—cellular level—link between innate and adaptative immune response: the initial triggers (viral infections, UV light exposure, silica dust) release cellular debris from apoptotic cells, exposing Nucleic Acids (NA) and starting production of type I IFN from plasmacytoid Dendritic Cells (pDCs). When acting on a specific genetic background, a vicious circle will begin, with continue activation of pDCs and further release of IFN. Type I IFN acts as an immunomodulator, primming DCs to a potent antigen-presenting cells—mature DC (mDCs), and this will induce differentiation of naïve CD 4 ⁺ T cells (LfT CD4) and appearance of CD 8 ⁺ memory T cells (LfT CD8), with cytokine production and tissue inflammation and exposer of auto-antigens (auto Ag). B-cells function is also influenced by type I IFN, leading to an increased survival and activation, including differentiation and class-switch recombination, causing autoantibody (autoAb) production. B. Schematic representation of IFN response in multifactorial SLE and in monogenic diseases driven by IFN, emphasising common pathway, but different starter: multifactorial versus monogenic defect related to nucleic acid handling—molecular level: IFN production is normally induced by viruses, bacteria, or microbial nucleic acids via recognition through Patterns Recognition Receptors (PRRs). IFN binds to transmembrane receptor—IFNAR, followed by activation of Janus kinase (JAK) 1 and tyrosine kinase (TIC) 2 pathway with phosphorylation of signal transducer and activator of transcription (STAT) 1, STAT 2 proteins, and formation of interferon regulatory factor (IRF) 9. This will promote IFN—stimulated genes (ISG) responsible for action on both innate and adaptative immune response.

Consequence of over-production of IFN will be visible not only on other genes, but also on most cells of both immune responses, innate and adaptative. Type I IFN acts as an immunomodulator, primming DCs to a potent antigen-presenting cells, being able to induce differentiation of naïve CD 4 ⁺ T cells and also development of CD 8 ⁺ memory T cells. An expansion of autoreactive T cells will be possible since IFN will increase the differentiation of Th17 cells and supress Treg function [26, 39, 40]. B-cell function is also influenced by type I IFN, leading to an increased survival and activation, including differentiation and class-switch recombination, causing autoantibody production. Antibody production is also stimulated by the action of type I IFN on monocytes, increasing the production of B cell activating factor [41, 42]. Schematic representation of IFN pathway, both on cellular (Figure 2A) and molecular (Figure 2B) levels, can be found in Figure 2.

### 3.3 IFN perspective in SLE pathogenesis

The initial trigger—like viral infections, UV light exposure, silica dust—will induce synthesis of small amounts of type I IFN, releasing cellular debris from apoptotic cells. In a patient with genetic background that predispose to SLE, loss of tolerance will increase expression of autoantigens from dying cells. These will represent the signal for production of autoantibody and appearance of immune complexes (ICs) and will further stimulate production of type I IFN at DCs level. A vicious circle will start, since the ICs and altered clearance of the apoptotic cell will continue stimulate IFN loop via TLRs, maintaining active both autoimmunity and inflammation at targeted sites, leading to irreversible damage in time [26]. Although a cytokine of the innate immunity, IFN represents a link between innate and adaptative immune response. SLE patients with high IFN activity will have increase titre of anti—double stranded DNA (dsDNA) antibody (ab), anti-Sjogren'syndrome-related antigen A (SSA) and anti-ribonucleoprotein (RNP) ab, highlighting the connection of IFN with B-cell maturation and autoantibody production [43].

## 4 Clinical rationale: IFN perspective

### 4.1 SLE and interferonopathies: two different pathologic conditions marked with multiple overlaps

Part of the autoinflammatory disease, the interferonopathies represent a group of rare monogenic disorders characterised by the overproduction of type I IFN as a result of mutations in genes responsible of handling of nucleic acids [26, 29]. According to the 2017 classification of the International Union of Immunological Societies, 13 types of interferonopathies were recognized until now [44]. Being monogenic diseases, symptoms start at an early age with different grade of severity, according to the penetrability of the genetic defect [45]. Pathogenic role of IFN was demonstrated and associated with different clinical phenotype with the help of interferonopathies. These patients exhibit fever, flu-like symptoms, cutaneous involvement like rash, photosensitivity, alopecia, chilblain accrual lesions or livedo reticularis, neurologic involvement with encephalitis, basal ganglia calcifications secondary to microangiopathies, hemiparesis, aphasia, psychiatric disorder. In some cases, patients may display feature of autoimmunity, which is consistent with the prominent role of type I IFN in driving autoantibody production, displaying evanescent, low—titre autoantibodies, resembling an autoimmune disease like SLE [46, 47]. Modulating IFN signalling by blocking JAK-STAT pathway may reduce the clinical manifestations in these patients [26]. Interferences with SLE clinical picture are found in Aicardi-Goutières syndrome, Sting- Associated Vasculopathy with onset in Infancy (SAVI) and monogenic Systemic Lupus Erythematosus.

### 4.2 Aicardi-Goutières Syndrome

The prototype of the interferonopathies is considered Aicardi-Goutières Syndrome. It was first described in 1984, when eight children were diagnosed with early-onset encephalopathy of unknown origin and labelled as pseudo-TORCH (toxoplasmosis, rubella, cytomegalovirus and herpes) since they were serologically negative for infections. The term interferonopathy was attributed in 2003, trying to underline the resemblance with a viral infection, suggesting a common pathogenic disturbance: upregulation of IFN—α activity [48, 49]. It is inherited in an autosomal recessive manner and seven subtypes were described until now, according to seven gene mutations identified [50]. Although neurologic involvement is the most characteristic, the pathognomonic extra-neurologic sign is cutaneous vasculopathy represented by the chilblain—like lesions mostly on the fingers and toes. More than half of the patients will exibit an autoimmune phenotype with haemolytic anaemia and SLE-like autoantibodies [51].

### 4.3 Sting- Associated Vasculopathy with onset in infancy

SAVI is another type *I interferonopathy* with skin and lungs involvement, produced by monogenic STING gain—of—function mutation, with skin involvement ranging from mild chilblain vasculopathy to digital amputations, nasal septum perforation, livedo reticularis, facial rash, periungual erythema, onychodystrophy [52, 53]. Respiratory tract involvement is represented by interstitial lung disease with progressive fibrosis [54]. High titre antinuclear antibodies (ANA), positive rheumatoid factor, or anti-neutrophil cytoplasmic antibodies might be expressed by these patients [55, 56].

### 4.4 Monogenic systemic lupus erythematosus

Childhood onset SLE is linked to a single gene mutation, defining monogenic or Mendelian SLE. At least three classes of gene mutations have been described [57, 58]. The most frequent form is associated with mutation at the level of the complement system (mostly C1q and C4) leading to a defective apoptosis [57]. The other two forms involve directly IFN pathway. Recently, development of a Mendelian SLE was associated with an endonuclease gene mutation, leading to a DNAse 1 deficiency. Excess of extracellular apoptotic materials, including nucleic acids expressed by microparticles, will be sensed by DAMPs, contributing to the production of type I IFN. Patients with DNAse 1 deficiency will have early onset nephritis, cutaneous involvement like urticarial vasculitis and high titre of multiple autoantibody (ANA, dsDNA). More recent, a type *I interferonopathy* secondary to DNAse2 deficiency was described. These patients exibit a typical clinical picture, with neonatal hepatosplenomegaly, cholestatic hepatitis and pancytopenia, membrano-proliferative glomerulonephritis, deforming arthropathy, foot vasculitis and constant high titre of anti-ds DNA antibody [59, 60]. A third subtype of monogenic SLE is direct related to IFN signal, being associated with genes encoding the enzymes involved in the endogenous nucleic acid degradation. It involves TREX1 mutations in an autosomal dominant form and it is related to early-onset familial chilblain lupus [6, 57, 61, 62]. This is characterised by the appearance of cold-induce vasculitis and might be associated with neuro-psychiatric manifestations: hemiparesis, facial paralysis, aphasia, hemianopsia, psychiatric disorders [63].

### 4.5 What can be learn from crosstalk between autoimmunity and autoinflammation via IFN?

Classical SLE, as polygenic pathology is associated with an IFN signature. Interferonopathies as monogenic disorders show autoimmune features, demonstrating once again that IFN is an important link between autoimmunity and autoinflammation. Figure 2B highlights the common pathogenic pathway with different starter: multifactorial defect in classical SLE versus monogenic defect in genetic interferonopathies.

Recognised only in the last decade, a huge step was done concerning monogenic interferonopathies, allowing to accumulate important evidence concerning pathogenesis or clinical aspects. Moreover, a step forward became possible about treatment to, with data accumulating from clinical case reports and observational studies. No doubt, knowledge gathered from autoimmune diseases—including SLE—contributed to that. This knowledge should work both ways. Although rare diseases, usually diagnosed in paediatric population, awareness of them will contribute to recognition of IFN implication in multifactorial, adult SLE. We are in an era of a more precise medicine. In real life settings, these newly described, rare disorders could help the clinician to associate specific manifestations of polygenic SLE to an IFN predominance pathway. This is even more important since the evaluation for IFN signature status is still difficult to perform in a daily basis [26, 27]. Table 2 emphasise congruent points between adult, multifactorial SLE and monogenic disease driven by IFN.

**Table 2:** Features of monogenic diseases driven by IFN that can be found in adult, multifactorial SLE.

Common features with multifactorial SLE	Aicardi-Goutières Syndrome	SAVI	Monogenic SLE DNAse subtype	Monogenic SLE TREX1 subtype
Clinical features	- Fevers, malaise - Chilblains vasculopathy - Skin rash, oral ulceration, Raynaud - Arthritis, - Glomerulonephritis, - CNS involvement with encephalopathy, seizures, neurocognitive impairment	- Fevers, malaise - Chilblains vasculopathy - Skin rash, livedo reticularis, onychodystrophy - Interstitial lung disease	- Fevers - Urticarial vasculitis - Arthritis - Glomerulonephritis - Cholestatic hepatitis	- Fevers, malaise - Chilblains vasculopathy - photosensitivity, Raynaud, oral ulceration, - Glomerulonephritis, - CNS involvement with stroke, hemiparesis, facial paralysis, aphasia, hemianopsia, psychiatric involvement
Laboratory features	- Thrombocytopenia, leukopenia, haemolytic anaemia - ANA, anti dsDNA ab - High level of IFN in cerebrospinal fluid	- ANA - antiphospholipid ab	- Pancytopenia - ANA, anti dsDNA ab	- Pancytopenia - ANA
References	48–51	52-56, 66	59, 60	6, 57, 61-63

Different clinical and laboratory abnormalities of SLE are now recognised to be associated with IFN involvement, as flu-like symptoms, muscle and joint pain, fever, serositis, headache, fatigue or cytopenia related to the suppressive effect of IFN on bone marrow [64, 65].

As highlighted already, monogenic interferonopathies often present with cutaneous involvement, including malar rash and alopecia [66], associated with renal or CNS involvement [16]. There are data that link these types of organs involvement with high IFN also in SLE. IFN signature is correlated with clinical cutaneous disease activity as measured by the Cutaneous Lupus Erythematosus Diseases Activity Score (CLASI) [67]. Clinical trials showed improvement of skin manifestations in SLE patients by blocking IFN signalling at different level—pDC, receptor, JAK-STAT pathway. All had favourable effect on skin lesions [68, 69, 70]. JAK blockade was found to work also for skin manifestations in patients with interferonopathies [71].

In paralel with what we learn from clinical pictures of interferonophaties, renal and CNS involvement of SLE patients are corelated with a strong IFN signature. IFN is implicated in both the inflammatory process and in the development of damage in SLE nephritis. Increased levels of type I IFN have been found in cerebrospinal fluid of lupus patients with neuropsychiatric (NP-SLE) complaines. Renal and NP-SLE are among the most severe, yet the most deprived from the targeted treatment perspective since the clinical trials are scarce. IFN inhibition might be a suitable treatment for these severe organ involvement in SLE and data accumulated from monogenic interferonopathies could offer an argument for designing trials that are specific for severe organ involvement [26, 72].

## 5 Treatment rationale: IFN perspective

### 5.1 Stratyfing SLE patients according to clinical phenotype

Personalised medicine is a target nowadays even for multifactorial diseases like SLE, but biomarkers or genetic evaluation are difficult to perform in real life. Currently there is no standardisation about methods, timing, or even results interpretation when trying to assess different pathogenic pathways in clinical settings and this is also the case for type I IFN.

Regarding IFN, available data from genome-wide expression analysis suggests that 50–80 % from adult SLE and up to 90 % of paediatric SLE overexpress IFN signature [26, 73]. High IFN expression is associated with more severe disease, with longitudinal studies showing a stable pattern across time [73, 74].

Published in 2022, largest longitudinal study until now that investigated IFN status in adult SLE outlined the importance of splitting patients in high IFN versus low IFN subgroup rather than following them timely—dependent or activity—dependent from the IFN perspective. Stability of IFN over time independently of the disease activity at some point or standard of care treatment administration (low-medium dose of glucocorticoids, Hydroxychloroquine or immunosuppressives) was an important finding [73]. It suggests that patients from high IFN subgroup have active the vicious, continuum IFN loop as shown in Figure 2A and this make them candidates of more targeted, now available anti—IFN treatment [75]. Moreover, baseline or stable high IFN subgroup (with “stable high IFN” meaning multiple evaluation in different time points) patients had significantly more active disease opposite to low IFN subgroup, with disease activity been related to skin, articular, haematological, serological or renal involvement [73]. These findings outline resemblance with clinical picture of interferonopathies (Table 2).

Splitting patients in these subgroups outside the clinical trials is not so easy, measurement of IFN in biological samples being still a challenge. There are indirect methods to assess IFN I, but with limited applicability because of cost, complexity, or availability, like IFN I activity assay, IFN signature or more recently developed single molecule array (SIMOA) [75]. Identification of specific clinical aspects learned from monogenic diseases could offer a suitable alternative for stratification of SLE patients in daily practice aiming for a better care.

### 5.2 IFN perspective upon SLE treatment

Standard of care therapies for patients with SLE are represented by glucocorticoids, antimalarial and immunosuppressants and they are used according to 2019 recommendations based on expert opinion and literature reviews [76]. Although usually associated with favourable response at least on short term, some patients might experience a partial response or might be non—responders. Moreover, side effects mainly related to glucocorticoids will contribute to damage accrual, making the need for a more targeted treatment more acute.

A new perspective started with the era of biologics. A wide spectrum of molecules was evaluated or is under evaluation, but with such a complex disease, study validation to emerge in therapy approval was difficult to achieve. This is way, a multitude of abnormal pathogenic pathways that looked like a valid target failed to perform. Only two biologics are now approved for SLE patients, with one being IFN target therapy—Anifrolumab [76, 77].

Anifrolumab is a fully humanised Immunoglobulin Gk1 (IgG) monoclonal ab that blocks all type I IFN/IFN receptor signalling, supressing the IFN signature gene and interrupting the vicious loop of IFN that trigger activation of both innate and adaptative immune response [75, 77]. It is indicated for adult SLE patients with moderate/severe active disease despite receiving standard treatment. A favourable effect on glucocorticoid tapering was also observed and this could represent a chance to reduce the damage accrual [77].

Approved since 2021, there still unanswered questions: when, to whom or for how long to be given. If we take into consideration the high persistent IFN subgroup patients these are the patients that should benefit most and not only during active disease, but in any moment when the IFN continuum vicious circle is active. SLE patients suffer not only from active disease, but also from irreversible damage related to uncontrolled inflammation and this should be taken into consideration when designing future trials. IFN blockade in SLE represents now an interesting perspective. Many trials that target this pathogenic pathway are under evaluation and they should investigate also the clinical phenotype of patient expected to respond to such therapies.

## 6 Conclusions

Multifactorial SLE is one of the hardest to treat systemic disorder. It represents more than a Lf B disease, with innate immunity having also an important role both for disease initiation and perpetuation. IFN pathway connects innate to adaptative immune response that will disseminate in a vicious loop, being responsible for tissue inflammation and contributing to Lf B action and autoantibody production. Being able to recognise an IFN phenotype learned from interferonopathies could improve real life evolution of SLE patients. Choosing the right treatment according to a possible pathogenic pathway predominance will increase the chance for a personalized treatment even for patients with complex diseases like multifactorial SLE.

## Declarations

### Author contribution statement

All authors listed have significantly contributed to the development and the writing of this article.

### Funding statement

This research did not receive any specific grant from funding agencies in the public, commercial, or not-for-profit sectors.

### Data availability statement

Data will be made available on request.

### Declaration of interest's statement

The authors declare no conflict of interest.

### Additional information

No additional information is available for this paper.
